# Diagnostic efficacy of sonographic measurement of laryngeal air column width difference for predicting the risk of post-extubation stridor: A meta-analysis of observational studies

**DOI:** 10.3389/fmed.2023.1109681

**Published:** 2023-01-19

**Authors:** Wen-Wen Tsai, Kuo-Chuan Hung, Yen-Ta Huang, Chia-Hung Yu, Chien-Hung Lin, I-Wen Chen, Cheuk-Kwan Sun

**Affiliations:** ^1^Department of Education, Chi Mei Medical Center, Tainan City, Taiwan; ^2^School of Medicine, College of Medicine, National Sun Yat-sen University, Kaohsiung City, Taiwan; ^3^Department of Anesthesiology, Chi Mei Medical Center, Tainan City, Taiwan; ^4^Department of Surgery, College of Medicine, National Cheng Kung University Hospital, National Cheng Kung University, Tainan City, Taiwan; ^5^Department of Anesthesiology, Chi Mei Medical Center, Liouying, Tainan City, Taiwan; ^6^Department of Emergency Medicine, E-Da Hospital, I-Shou University, Kaohsiung City, Taiwan; ^7^School of Medicine for International Students, College of Medicine, I-Shou University, Kaohsiung City, Taiwan

**Keywords:** air column width difference, post-extubation stridor, tracheal extubation, ultrasound, meta-analysis

## Abstract

**Background:**

This meta-analysis aimed at assessing the diagnostic accuracy of ultrasound-measured laryngeal air column width difference (ACWD) in predicting post-extubation stridor (PES) in intubated adult patients.

**Methods:**

We searched the Medline, Cochrane Library, EMBASE, and Google scholar databases from inception to October, 2022 to identify studies that examined the diagnostic accuracy of ACWD for PES. The primary outcome was the diagnostic performance by calculating the pooled sensitivity, specificity, and area under the curve (AUC). The secondary outcomes were the differences in ACWD and duration of intubation between patients with and without PES.

**Results:**

Following literature search, 11 prospective studies (intensive care setting, *n* = 10; operating room setting, *n* = 1) involving 1,322 extubations were included. The incidence of PES among the studies was 4–25%. All studies were mixed-gender (females: 24.1–68.5%) with sample sizes ranging between 41 and 432. The cut-off values of ACWD for prediction of PES varied from 0.45 to 1.6 mm. The pooled sensitivity and specificity of ACWD for PES were 0.8 (95% CI = 0.69–0.88, *I*^2^: 37.26%, eight studies) and 0.81 (95% CI = 0.72–0.88, *I*^2^: 89.51%, eight studies), respectively. The pooled AUC was 0.87 (95% CI = 0.84–0.90). Patients with PES had a smaller ACWD compared to those without PES (mean difference = −0.54, 95% CI = −0.79 to −0.28, *I*^2^: 97%, eight studies). Moreover, patients with PES had a longer duration of tracheal intubation than that in those without (mean difference = 2.75 days, 95% CI = 0.92, 4.57, *I*^2^: 90%, seven studies).

**Conclusion:**

Ultrasound-measured laryngeal ACWD showed satisfactory sensitivity and specificity for predicting PES. Because of the limited number of studies available, further investigations are needed to support our findings.

**Systematic review registration:**

https://www.crd.york.ac.uk/prospero/, identifier CRD42022375772.

## 1. Introduction

Endotracheal intubation is one of the most crucial life-saving procedures to support the respiratory system for critically ill patients on ventilators. Airway complications can, however, arise from tracheal intubation. Although intubation-related airway complications are usually mild in the operating room setting in which the patients receive a short-term intubation (1), prolonged intubation can cause laryngeal edema, increased airflow resistance, and partial airway obstruction that could contribute to post-extubation stridor (PES) (2–4). The development of PES, which occurs in 1.5–26.3% of patients in the critical care setting (4–6), is likely to be associated with an increased risk of respiratory failure and reintubation (6, 7), which is associated with prolonged intensive care unit (ICU) stay, morbidity, and mortality (5).

Recent studies have examined the use of ultrasound-guided techniques, which allows visualization of vocal cords and larynx, to assess airway patency (8–11). The laryngeal air column width, which refers to the width of the acoustic shadow at the level of the vocal cords, can be measured with ultrasonography (12). The laryngeal air column width difference (ACWD), which is defined as the difference in width of the air column between balloon cuff inflation and deflation, has been reported to be a tool for predicting PES (4). Previous studies have demonstrated a significantly smaller ACWD in patients with PES compared to those without (13, 14). Nevertheless, the estimated sensitivity and specificity for predicting PES varied widely among the literature, namely, 50–97% and 57–90%, respectively (9, 13, 14), suggesting notable discrepancy in technical skills involved in the ultrasound assessment of laryngeal edema. Taking into account the lack of evidence addressing this issue, this meta-analysis aimed primarily at investigating the pooled diagnostic efficacy [i.e., pooled sensitivity, specificity, and data derived from the area under receiver operating characteristic (ROC) curve] associated with the use of ultrasound ACWD measurement for predicting PES in intubated patients with planned extubation. We also compared the ACWD and duration of tracheal intubation between patients with and those without PES (i.e., secondary outcomes).

## 2. Materials and methods

### 2.1. Study protocol

This systematic review, which followed the preferred reporting items for a systematic review and meta-analysis of diagnostic test accuracy studies (PRISMA-DTA) guideline, was registered at PROSPERO (CRD42022375772).

### 2.2. Data source and literature search

Two authors independently searched the databases including Medline, Cochrane library, Embase, and Google scholar to identify eligible studies focusing on sonographic measurement of ACWD in the prediction of PES from inception to 14, November, 2022, without language restriction. The following keywords were used for literature search: (“Post-extubation” or “Tracheal extubation” or “Airway extubation*” or “Intratracheal extubation*” or “Endotracheal extubation*”) and (“Ultrasound” or “Ultrasonography” or “Ultrasound-guided” or “Sonography” or “Echography” or “Echotomography” or “Ultrasonic”” or “Laryngeal ACWD”) and (“Stridor” or “Airway edema” or “Airway obstruction” or “subglottic stenosis” or “Laryngeal edema”). Manual searching was also performed for discovering potentially relevant studies. Conflicts between authors were solved by consensus that involved a third investigator. The search detail for one of the databases (i.e., Medline) is shown in Supplementary Table 1.

### 2.3. Inclusion and exclusion criteria

Studies were included if they met the following criteria: (a) adult patients in whom tracheal extubation was attempted in the critical care setting or operating room setting; (b) sonographic measurement of ACWD was used to predict laryngeal edema or PES regardless of cut-off value. The criteria for the diagnosis of laryngeal edema/PES were based on those of individual studies; (c) availability of details regarding sensitivity, specificity, number of patients with or without PES, and the value of ACWD. For the current meta-analysis, randomized control trial, cohort study, and case-control study were all considered eligible for inclusion.

Excluded studies were (a) those reported as abstracts, conference papers, case reports, case series, or review articles; (b) those focused on the pediatric population; (c) those without outcomes of interest; (d) those with no available full text; or (e) those that involved the combination of different tests to predict PES.

### 2.4. Data extraction

Data was independently extracted from the individual studies by two authors. Disagreements were resolved by a third investigator. The following data were collected: first author’s name, study characteristics (e.g., sample size, setting), patient’s age, gender, sensitivity, specificity, value of ACWD, duration of tracheal intubation, incidence of PES, and country. If different cut-off values of ACWD were used to predict PES in one study, we extracted the dataset with the highest area under the curve (AUC) available. For missing information, we tried to contact the authors of the articles.

### 2.5. Outcomes and definitions

The main outcome was the diagnostic efficacy of sonographic measurement of ACWD in predicting PES. The pooled sensitivity, specificity, and ROC were used to assess the diagnostic efficacy regardless of the threshold used. The secondary outcomes included the differences in ACWD and the duration of tracheal intubation between patients with and those without PES. ACWD was defined as the use of ultrasound to measure ACWD between the inflated and deflated cuffs regardless of the manufacturer of the ultrasound equipment. The diagnostic criteria of PES were in accordance with those of individual studies.

### 2.6. Quality assessment

The quality of each included study was evaluated using the Quality Assessment for Diagnostic Accuracy Studies-2 (QUADAS-2) tool that comprises two categories, namely, “risk of bias” and “applicability concerns.” While the former contains four domains, the latter consists of three domains for quality assessment (15). Two authors subjectively reviewed all the included studies and rated each domain as “low risk,” “some concerns,” or “high risk.” Disagreements were resolved through discussion till a consensus was reached. A third author was involved if necessary.

### 2.7. Statistical analysis

The association of PES with ACWD and the duration of intubation was analyzed by using a random effects model with Review Manager version 5.3. We calculated the pooled estimates of sensitivity and specificity as well as the positive and negative likelihood ratios [LR (+) and LR (−)], which were acquired with the formulas: LR (+) = sensitivity/(1−specificity); LR (−) = (1−sensitivity)/specificity. While statistical heterogeneity was investigated using Cochran Q-statistics, heterogeneity between studies was evaluated with the random effects model using the *I*^2^ statistics. The diagnostic performance of sonography was assessed with AUC from constructed summary ROC (sROC) curves (16). Based on LR (+) and LR (−), post-test probability was estimated with a Fagan’s nomogram. The potential publication bias was examined by inspecting Deek’s funnel plot. A *p* < 0.05 was regarded as statistically significant. Forest plots of pooled estimates of sensitivity and specificity, Deek’s funnel plot, sROC curve, and Fangan’s nomogram plot were constructed with the meta-analytical integration of diagnostic test accuracy studies (MIDAS) command in Stata 15 (StataCorp LLC., College Station, TX, USA) as previously reported (17).

## 3. Results

### 3.1. Selection and characteristics of studies

**FIGURE 1:**
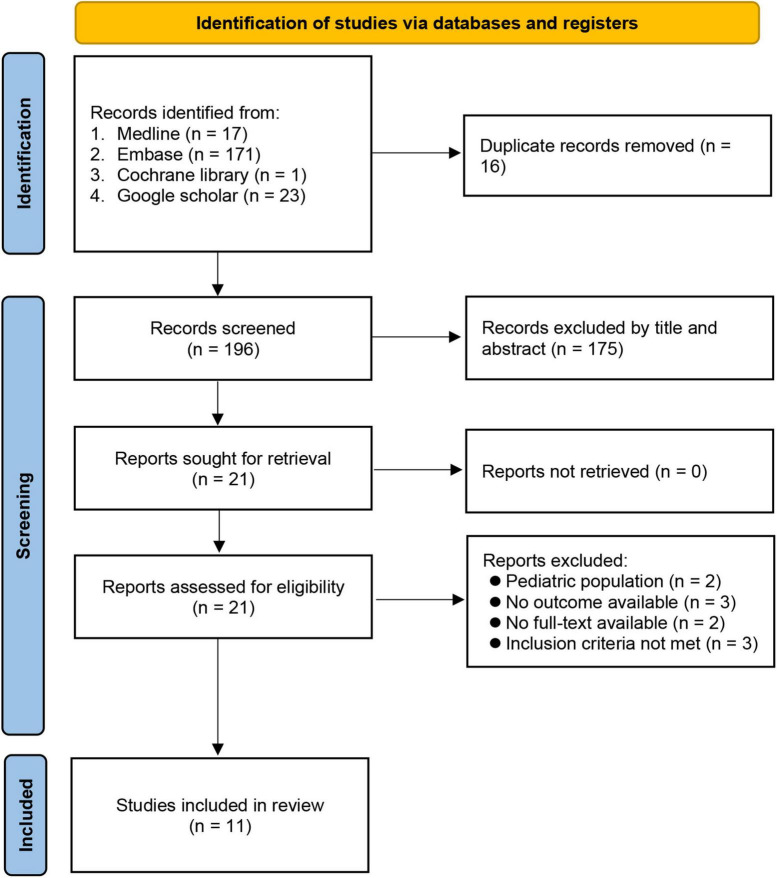
PRISMA flow diagram of study selection for the current meta-analysis.

A literature search identified 212 potentially eligible articles. After article review, 16 duplicates were removed. Of the remaining 196 articles screened based on title and abstract, 175 were excluded. After eliminating 10 more articles according to our exclusion criteria, 11 studies involving 1,322 participants were included for the current analysis (9, 13, 14, 18–25) (Figure 1). The characteristics of the included studies published between 2013 and 2021 are shown in Table 1. Of the 11 studies, eight provided details for the calculation of sensitivity and specificity (9, 13, 14, 18, 19, 22, 23, 25). Eight (9, 14, 18–23) and seven studies (9, 14, 18, 20, 21, 23, 24) were available for analysis of the association of PES with ACWD and the duration of intubation, respectively. Most studies (i.e., 10) were conducted prospectively in the intensive care unit setting (9, 13, 14, 18, 20–25) and one was performed in the operating room setting (19). All studies were mixed-gender (females: 24.1–68.5%) with sample sizes ranging from 41 to 432. The cut-off values of ACWD for the prediction of PES were available in nine studies (range, 0.45–1.6 mm). The incidence of PES was 4–25%. Six studies were conducted in Egypt (9, 14, 18, 20, 22, 25), two in India (19, 24), one each in the USA (21), Thailand (23), and Iran (13).

**TABLE 1:** Characteristics of studies (*n* = 11).

References	Number of patients	Female (%)	Mean age (year)^§^	Setting	ACWD (mm)^§^	Cut-off value (mm)	Duration of intubation (days)^§^	PES (%)	Country
Abd Elghafar et al. (18)	70	35.7	39 vs. 38	ICU	1.14 ± 0.70 vs. 1.78 ± 0.53	1.5	7.78 vs. 4.25	12.9	Egypt
Bhargava et al. (19)	200	68.5	46 vs. 45	OR	0.13 ± 0.03 vs. 0.29 ± 0.026	1	140 vs. 130^‡^	6	India
El-Baradey et al. (9)	432	26.9	25.4 vs. 47.3	ICU	0.6 ± 0.5 vs. 1.9 ± 0.7	0.9	14.3 vs. 9.6	10.4	Egypt
Hasan and Ahmed (20)	58	24.1	53.1 vs. 55.2	ICU	0.56 ± 0.07 vs. 0.86 ± 0.5	NA	7.23 vs. 6.53	12.1	Egypt
Mikaeili et al. (13)	41	36.6	57.2⁋	ICU	NA	0.85	At least 24 h	9.8	Iran
Mohammed et al. (14)	167	37.7	59.5 vs. 56.2	ICU	0.541 ± 0.33 vs. 1.237 ± 0.44	0.65	10.6 vs. 5.9	10.2	Egypt
Patel et al. (21)	51	41.2	NA^†^	ICU	0.4 ± 0.2 vs. 0.8 ± 0.7	0.45	3.5 vs. 3.9	4	USA
Sahbal et al. (22)	50	38.0	NA	ICU	0.95 ± 0.08 vs. 0.97 ± 0.09	0.905	At least 24 h	8	Egypt
Sutherasan et al. (23)	101	38.6	72.2 vs. 66.9	ICU	1.08 ± 0.81 vs. 1.99 ± 0.79	1.6	7.9 vs. 6.2	16.8	Thailand
Venkategowda et al. (24)	72	48.6	54 vs. 55	ICU	NA	NA	5.6 vs. 3.9	6.9	India
Zytoun et al. (25)	80	42.5	46.4⁋	ICU	NA	0.9	40% vs. 55%^#^	25	Egypt

Operating room (total thyroidectomy); PES, post-extubation stridor; ⁋overall population; ^†^<45 y/o: n = 13; 45–54 y/o: n = 13; >55 y/o: n = 25; ^§^presented as PES vs. non-PES; ACWD, air column width difference; ^‡^minutes; NA, not available; ^#^proportion of patients (PES vs. non-PES) with duration of tracheal intubation ≥5 days.

**FIGURE 2:**
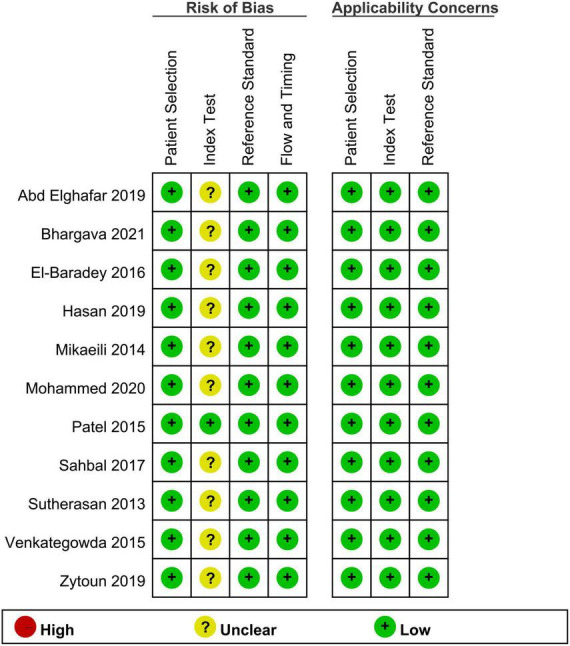
Risk of bias and applicability concerns.

The risk of bias and applicability concerns in all studies are shown in Figure 2. Regarding the risk of bias, the domains of patient selection, reference standard, flow, and timing were considered low in all studies. However, the risk of bias on the domain of index test was deemed unclear in 10 of the studies due to their lack of a pre-defined cut-off value of ACWD. For the applicability concerns, all studies are considered at low risk of bias.

### 3.2. Diagnostic efficacy of ultrasonography for prediction of post-extubation stridor

**FIGURE 3:**
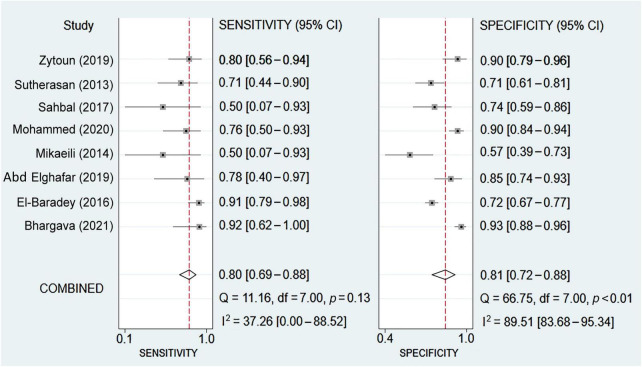
Forest plot showing the pooled sensitivity and specificity of sonography-measured air column width difference (ACWD) in predicting post-extubation stridor (PES).

**FIGURE 4:**
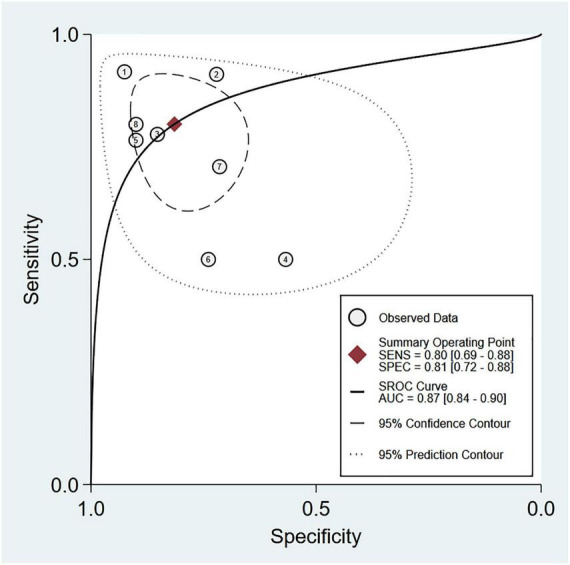
Summary receiver operating characteristic (sROC) curve analysis of sensitivity and specificity of air column width difference (ACWD) for predicting post-extubation stridor (PES). Weighted sROC is shown as a solid line. Open circles denote estimates of sensitivity and (1–specificity) of individual studies. Diamonds represent pooled point estimates of outcomes. AUC, area under the curve; SENS, sensitivity; SPEC, specificity.

**FIGURE 5:**
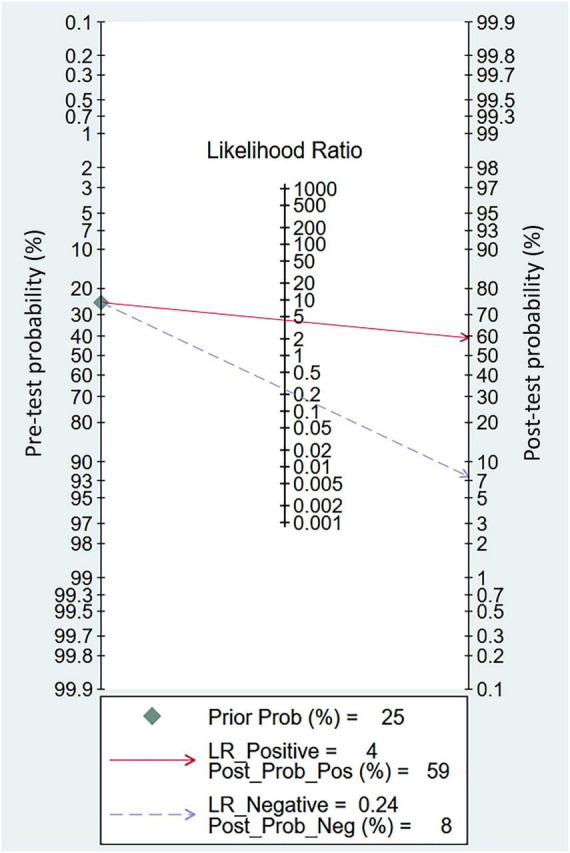
Fagan’s nomogram plot for assessing clinical utility of sonography-measured air column width difference (ACWD) in post-extubation stridor (PES) prediction. LR, likelihood ratio; Prob, probability; Pos, positive; Neg, negative.

**FIGURE 6:**
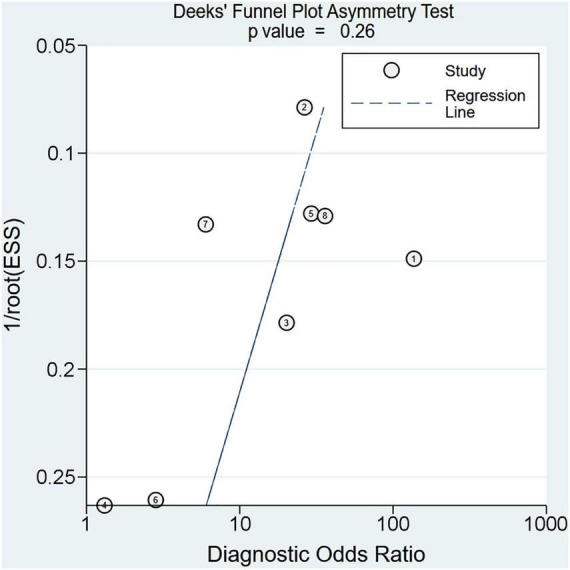
Deek’s funnel plot asymmetry test for assessment of publication bias across the included studies.

Eight studies were available for the calculation of the sensitivity and specificity (9, 13, 14, 18, 19, 22, 23, 25). Relevant data and the definitions of PES are summarized in Supplementary Table 2 and Supplementary Table 3, respectively. The pooled sensitivity and specificity were 0.8 (95% CI = 0.69–0.88, *I*^2^: 37.26%) and 0.81 (95% CI = 0.72–0.88, *I*^2^: 89.51%), respectively (Figure 3). The pooled AUC was 0.87 (95% CI = 0.84–0.90) (Figure 4). LR (+) and LR (−) were 4 and 0.24, respectively (Figure 5). The Fagan plot is shown in Figure 5. The Deek’s funnel plot asymmetry test showed a low risk of publication bias (*p* = 0.26; Figure 6).

### 3.3. The differences in air column width difference and duration of tracheal intubation in patients with and without post-extubation stridor

**FIGURE 7:**
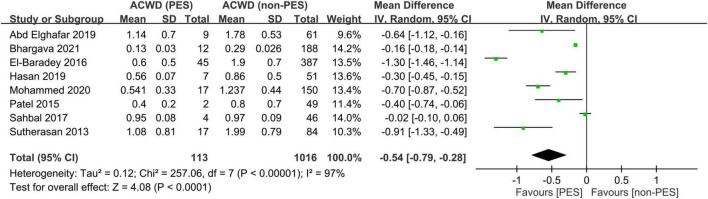
Forest plot comparing the air column width difference (ACWD) between patients with and without post-extubation stridor (PES). CI, confidence interval.

Eight studies including 1,129 participants were available for the analysis of ACWD between patients with and those without PES (9, 14, 18–23). By adopting a random-effects model, patients with PES had a smaller ACWD compared to those without PES (mean difference = −0.54, 95% CI = −0.79 to −0.28, *I*^2^: 97%) (Figure 7). Sensitivity analysis with the leave-one-out method revealed consistent findings in support of the strength of evidence.

**FIGURE 8:**
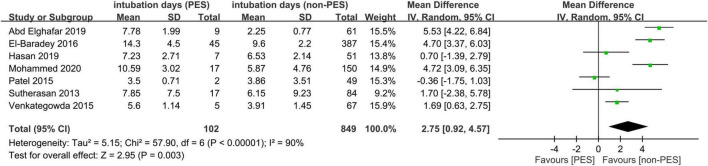
Forest plot comparing intubation time between patients with and without post-extubation stridor (PES). CI, confidence interval.

Seven studies with 951 participants provided details for the analysis of the association between the duration of tracheal intubation and PES (9, 14, 18, 20, 21, 23, 24). With a random-effects model, patients with PES showed a longer duration of tracheal intubation compared to those without (mean difference = 2.75 days, 95% CI = 0.92, 4.57, *I*^2^: 90%) (Figure 8). Sensitivity analysis demonstrated robustness of the finding.

## 4. Discussion

The negative impact of extubation failure and reintubation on prognostic outcomes (e.g., prolonged intensive care unit stay) in critically ill patients has underscored the importance of identifying those at high risks of PES (6). Our findings not only demonstrated a lower ACWD values (mean difference = −0.50) in patients with PES but also showed a favorable sensitivity (i.e., 80%) and specificity (i.e., 81%) of using ACWD as a predictor of this complication with an AUC of 0.87. In view of the simplicity of the technique, our results suggested the feasibility of using ACWD as a guidance to determine the suitability of extubation to enhance patient safety in the critical and post-operative care settings.

Although recent advances in critical care technology have significantly improved the prognosis and survival of patients receiving ventilator support (26), extubation failure still occurs in approximately 10–15% of patients (27). Previous studies have shown that extubation failure may be associated with higher rates of tracheotomies, increased mortality, prolonged stay in the intensive care unit, and impaired functional outcome after recovery (28–30). Therefore, avoidance of premature tracheal extubation plays a vital role in the delivery of critical care. On the other hand, delay in weaning from mechanical ventilation could contribute to complications such as nosocomial infections, ventilator-induced lung injury, and delirium (31, 32). In addition, prolonged mechanical ventilation imposes a heavy financial burden in the intensive care unit setting (33). Laryngeal edema, which often manifests with stridor after tracheal extubation, is one of the most important contributors to extubation failure in the critical care setting (34). In fact, a previous study attributed failed extubation to the presence of laryngeal edema in up to 38% of all cases (35). To avoid premature or delayed tracheal extubation, a diagnostic technique that can reliably predict laryngeal edema or PES with high sensitivity and specificity is needed.

Although previous investigations have identified a number of potential risk factors for PES, including the female gender, difficult or prolonged intubation, use of large-sized endotracheal tubes, and a high cuff pressure (7, 36–39), such predictors are too non-specific to predict the occurrence of laryngeal edema and/or stridor in the real-world clinical scenario. In contrast, one of the well-defined techniques to predict the absence of post-extubation laryngeal edema was the cuff leak test (7, 40, 41). Despite the recommendation of performing a cuff leak test among mechanically ventilated adults with a high risk of PES by the American Thoracic Society and American College of Chest Physicians (42), a previous meta-analysis involving 28 studies with 4,493 extubations revealed a sensitivity and specificity of cuff leak test for predicting the absence of laryngeal edema of only 0.62 and 0.87, respectively. The low sensitivity raised a concern that this test may mislead clinicians to believe in the presence of laryngeal edema that unnecessarily prolonged mechanical ventilation (6). In addition, although the cuff leak test is supposed to be performed when the patient is well sedated with full mechanical ventilatory support, its accuracy may be impaired in real-world practice that involves patients who are being weaned from mechanical ventilation (e.g., on pressure support ventilation or spontaneous breathing trial) (23). Furthermore, apart from the space between the tracheal wall and the cuff after endotracheal cuff deflation, the accuracy of the cuff leak test also depend on other factors such as the expiratory tracheal airflow, expiratory time, and air trapping from collapsing airway related to chronic obstructive pulmonary disease (43, 44).

In the current meta-analysis, the pooled sensitivity and specificity of sonography-guide ACWD technique for predicting PES were 0.8 and 0.81, respectively, supporting the feasibility of this technique in the critical care setting. Moreover, in contrast to the cuff leak test that requires mechanical ventilation, the ACWD approach only involves sonographic measurement that is more practical in the intensive care unit (45). Besides, previous studies have reported additional merits of the ACWD technique including safety, ease of image acquisition because of conspicuous landmarks (i.e., cricoid and thyroid cartilage) even in obese individuals as well as the short distance between the skin to vocal cords (23). The additional advantage is the simplicity of interpretation with a larger ACWD representing a lower chance of laryngeal edema (23). In the current meta-analysis, the value of ACWD was lower in patients with PES compared to those without, further validating the theory of its operation. The high heterogeneity in this outcome may be attributed to the variations in intubation duration, size of tracheal tube, patient gender, and the operator’s experience.

Our results showed an incidence of PES ranging from 4 to 25%, which is consistent with the findings of previous studies that reported an estimated PES incidence of 1.5–26.3% in the critical care setting (4–6). Regarding the duration of intubation, the present study demonstrated a longer intubation time in patients with PES compared to those without. The finding supported a positive association of prolonged intubation with the risk of PES as previously reported (5). One of our included studies that focused on patients receiving total thyroidectomy in the operating room setting revealed an incidence of PES up to 6% (19), highlighting that even short-term tracheal intubation (e.g., 130–140 min) may cause PES in the operating room setting. Accordingly, the use of sonography-measured ACWD may also be recommended for those undergoing head and neck surgeries before extubation regardless of the duration of intubation to enhance patient safety.

There were several limitations in the current meta-analysis. First, the absence of standardized methods to assess the severity of laryngeal edema (4) across our included studies may bias our findings. Similarly, the definition of PES used in the studies may have varied and blinding of assessment may not have been adequate in individual studies. Second, although a previous meta-analysis reported that the pooled sensitivity and specificity of the cuff leak test for reintubation rate were 0.66 and 0.88, respectively (34), we did not assess the impact of sonography-measured ACWD on the reintubation rate because of a lack of relevant information. Third, the focus of most of our included studies on patients in the intensive care unit may restrict the applicability of our findings in other clinical settings. In addition, our inclusion of only adult patients may not justify the extrapolation of our results to the pediatric population. Fourth, the effects of other potential confounders (e.g., size of tracheal tube or gender) that may influence the accuracy of sonography-measured ACWD were not investigated in the present study because of limited data availability. Fifth, the application of steroids, which was noted in over one-fourth of patients (25.5%, *n* = 13) within 24 h of endotracheal extubation in one of the included studies (21), may impede the evaluation of the diagnostic accuracy of ACWD for PES. Finally, potential variations in calibration and image quality of ultrasound machines from different manufacturers, operator’s experience, as well as the head and neck position (e.g., flexion, extension) of the patient may influence distance measurement with the technique (23). Therefore, further investigations are warranted to address these issues.

## 5. Conclusion

Our results demonstrated that the measurement of laryngeal ACWD with ultrasound may be a clinically useful tool in the assessment of laryngeal edema or the prediction of PES. The favorable sensitivity and specificity may support its routine use in patients at high risk of PES. Nevertheless, the use of this technique does not obviate the need for close patient monitoring to ensure the absence of post-extubation complications. Further multicenter studies with standardized definitions and cutoffs, ideally paired with an intervention, are necessary to define the role of ACWD in routine clinical practice.

## Data availability statement

The original contributions presented in this study are included in this article/Supplementary material, further inquiries can be directed to the corresponding authors.

## Author contributions

W-WT and K-CH: conceptualization. Y-TH: methodology and software. C-HY and K-CH: validation. K-CH and Y-TH: formal analysis. C-HL and W-WT: investigation. I-WC: resources. I-WC and K-CH: data curation. K-CH, W-WT, I-WC, and C-KS: writing—original draft preparation. K-CH, I-WC, and C-KS: writing—review and editing. C-KS: visualization and supervision. All authors have read and agreed to the published version of the manuscript.
