# Supplementary material for: Diagnostic efficacy of sonographic measurement of laryngeal air column width difference for predicting the risk of post-extubation stridor: A meta-analysis of observational studies
Source: Front Med (Lausanne). 2023 Jan 19;10:1109681. doi: 10.3389/fmed.2023.1109681 (PMC9893004; doi:10.3389/fmed.2023.1109681)
Supplement: Supplementary file 1 [file Data_Sheet_1.docx]

**Supplemental Table 1.** Search strategies for Medline

| Database | # | Search syntax |
| --- | --- | --- |
| **MEDLINE (Ovid)** | 1 | ("Post-extubation" OR "Tracheal extubation" OR "Airway extubatiion*" OR "Intratracheal extubation*" OR "Endotracheal extubation*").mp |
|  | 2 | exp "Airway Extubation"/ |
|  | 3 | ("Ultrasound" OR "Ultrasonography" OR "Ultrasound-guided" OR "Sonography" OR "Echography" OR "Echotomography" OR "Ultrasonic" OR "Laryngeal air column width difference").mp |
|  | 4 | exp "Ultrasonography"/ |
|  | 5 | ("Stridor" OR "Airway edema" OR "Airway obstruction" OR "subglottic stenosis" OR "Laryngeal edema").mp |
|  | 6 | exp "Airway Obstruction"/ OR exp "Laryngostenosis"/ |
|  | 7 | (1 OR 2) AND (3 OR 4) AND (5 OR 6) |

**Supplemental Table 2.** Characteristics of studies which provided data for calculation of the sensitivity and specificity (n = 8)

| Study (year) | Sample size | Adult or Children | PES (%) | Sensitivity/ specificity/PPV/ NPV/ /AUC | Cut-off value (mm) | Country | Comment: Is ACWD effective? | PubMed reference number (PMID) |
| --- | --- | --- | --- | --- | --- | --- | --- | --- |
| Abd Elghafar 2019 | 70 | Adult | 12.9 | 0.74/0.85/0.29/0.9/0.85 | 1.5 | Egypt | Yes | NA† |
| Bhargava 2021 | 200 | Adult | 6 | 0.917/0.926/0.44/0.99/NR | 1 | India | Yes | 35110144 |
| El-Baradey 2016 | 432 | Adult | 10.4 | 0.91/0.72/0.97/0.5/NR | 0.9 | Egypt | Yes | 27468680 |
| Mikaeili 2014 | 41 | Adult | 9.8 | 0.5/0.57/0.11/0.91/NR | 0.85 | Iran | Uncertain | 24753828 |
| Mohammed 2020 | 167 | Adult | 10.2 | 0.765/0.9/0.46/0.97/0.896 | 0.65 | Egypt | Yes | NA† |
| Sahbal 2017 | 50 | Adult | 8 | 0.5/0.739/0.143/0.944/NR | 0.905 | Egypt | Uncertain | NA† |
| Sutherasan 2013 | 101 | Adult | 16.8 | 0.706/0.702/0.324/0.922/0.823 | 1.6 | Thailand | Yes | 23806246 |
| Zytoun 2019 | 80 | Adult | 25 | 0.8/0.9/0.727/0.931/NR | 0.9 | Egypt | Yes | NA† |

PES: post-extubation stridor; † available from Google scholar; ACWD: Air column width difference; NA: not available; NR: not reported; PPV: Positive Predictive Value; NPV: Negative Predictive Value; AUC: area under curve

**Supplemental Table 3**. Definition of post-extubation stridor in studies which provided data for calculation of the sensitivity and specificity (n = 8)

| Study (year) | Definition of post-extubation stridor |
| --- | --- |
| Abd Elghafar 2019 | The presence of high-pitched inspiratory wheeze localized to trachea or the larynx and associated with respiratory distress that requiring medical intervention in the form of steroids, neubilized racemic epinephrine, non-invasive positive pressure ventilation and or reintubation. |
| Bhargava 2021 | Respiratory distress occurring immediately after extubation characterised by inspiratory sounds of whistling, wheezing or grunting. |
| El-Baradey 2016 | High-pitched inspiratory crowing sound heard in the upper airway requiring medical intervention within 24h of extubation and associated with respiratory distress. |
| Mikaeili 2014 | The presence of a high-pitched inspiratory wheeze requiring medical intervention. Associated with respiratory distress within 24 hours of extubation, PES was accompanied with a respiratory rate >30/minute or increase by >10/minute from baseline. |
| Mohammed 2020 | The presence of a high‑pitched inspiratory wheeze localized to the trachea or the larynx and associated with respiratory distress, usually requiring medical intervention. |
| Sahbal 2017 | Not reported |
| Sutherasan 2013 | Detection of stridor from physical examination and direct visualization from the Glidescope video laryngoscopy, which were performed immediately after extubation. The laryngeal edema was defined as erythematous swelling of vocal cords with or without mucosal ulceration. Laryngeal stridor was defined by presence of inspiratory rhonchi on vocal cord area. |
| Zytoun 2019 | The presence of a high-pitched inspiratory wheeze localized to the trachea or the larynx and was associated with respiratory distress, usually requiring medical intervention. |
